# Test‐retest reliability of EEG network characteristics in infants

**DOI:** 10.1002/brb3.1269

**Published:** 2019-03-25

**Authors:** Bauke van der Velde, Rianne Haartsen, Chantal Kemner

**Affiliations:** ^1^ Department of Experimental Psychology Helmholtz Institute, Utrecht University Utrecht the Netherlands; ^2^ Department of Developmental Psychology Utrecht University Utrecht the Netherlands; ^3^ Centre for Brain and Cognitive Development, Department of Psychological Science University of London Birkbeck London UK

**Keywords:** brain development, EEG, functional connectivity, graph theory, infants, test‐retest reliability

## Abstract

**Introduction:**

Functional Electroencephalography (EEG) networks in infants have been proposed as useful biomarkers for developmental brain disorders. However, the reliability of these networks and their characteristics has not been established. We evaluated the reliability of these networks and their characteristics in 10‐month‐old infants.

**Methods:**

Data were obtained during two EEG sessions 1 week apart and was subsequently analyzed at delta (0.5–3 Hz), theta (3–6 Hz), alpha1 (6–9 Hz), alpha2 (9–12 Hz), beta (12–25 Hz), and low gamma (25–45 Hz) frequency bands. Connectivity matrices were created by calculating the phase lag index between all channel pairs at given frequency bands. To determine the reliability of these connectivity matrices, intra‐class correlations were calculated of global connectivity, local connectivity, and several graph characteristics.

**Results:**

Comparing both sessions, global connectivity, as well as global graph characteristics (characteristic path length and average clustering coefficient) are highly reliable across multiple frequency bands; the alpha1 and theta band having the highest reliability in general. In contrast, local connectivity characteristics were less reliable across all frequency bands.

**Conclusions:**

We conclude that global connectivity measures are highly reliable over sessions. Local connectivity measures show lower reliability over sessions. This research therefore underlines the possibility of these global network characteristics to be used both as biomarkers of neurodevelopmental disorders, but also as important factors explaining development of typical behavior.

## 1 INTRODUCTION

The brain is a complex network consisting of highly interconnected regions. During early childhood, these networks develop at a rapid pace. Electroencephalography (EEG) can be used to study this early development of functional networks (Boersma et al., 2013; Orekhova et al., 2014). The high temporal resolution of EEG allows for the study of high‐frequency oscillatory brain activity, while the infant is relatively unrestricted in its movements. Synchronized oscillatory activity allows for optimized flow of information between two regions (Fell & Axmacher, 2011) and therefore studying oscillatory brain activity, either at rest or during a task, gives insight in underlying functional connectivity and brain networks. Oscillatory brain activity ranges from ultraslow oscillations (0.05 Hz) to fast transient oscillations (up to 500 Hz) (Buzsáki, 2004). Infant EEG has limited functionality in the detection of high‐frequency oscillations, as contamination with muscle‐induced high‐frequency artifacts is difficult to remove. Therefore, most developmental EEG researchers focus on slower oscillatory activity, including delta (0.1–3 Hz), theta (3–6 Hz), alpha (6–12 Hz), beta (12–25 Hz), and low gamma (25–45 Hz) bands. Functionally distinct networks can be found at these different frequency bands, which is most notably represented in the spatial scale of oscillatory synchrony, which ranges from several centimeters in slow oscillations (Schoffelen, 2005) to micrometers in ultrafast oscillations (Izhikevich, 2001).

Functional brain networks and characteristics have been used in the past to study differences between typical and atypical brain development. In autism spectrum disorder (ASD) for example, global connectivity (the averaged connection strengths of the whole brain network) tends to be deteriorated at lower frequencies, which is compensated by increased global connectivity at higher frequencies (Boersma et al., 2013; O'Reilly, Lewis, & Elsabbagh, 2017; Orekhova et al., 2014; Peters et al., 2013; Righi, Tierney, Tager‐Flusberg, & Nelson, 2014). Similarly in children with attention‐deficit hyperactivity disorder show an increase in frontal low alpha connectivity and a decrease in frontal high alpha connectivity (Murias, Swanson, & Srinivasan, 2007).

Comparing these networks on a global connectivity level has shown usefulness. However, to better understand the differences between these complex networks on a detailed level, a graph theoretical framework can be used (Albert & Barabási, 2002; Bullmore & Sporns, 2009), which simplifies the network into nodes (centers of information or—in the case of EEG connectivity—EEG sensors) and edges (connections between the nodes). With this mathematical approach, several metrics can be calculated describing certain aspects of a network. The most commonly used network metrics are the characteristic path length (Lw), the average clustering coefficient (Cw) and the small‐worldness index (SWI). The characteristic path length is the average shortest path length between all nodes in the network. A shorter characteristic path length generally indicates a higher global efficiency in networks. The average clustering coefficient describes the number of clusters in a network. Higher clustering generally indicates higher local efficiency in networks. Small‐world networks are networks in which both short path lengths and high clustering are present. As such, small‐worldness is calculated as the ratio between the normalized clustering coefficient and the normalized path length. All of these characteristics have been connected to several neurodevelopmental disorders, like ASD (Peters et al., 2013; Rudie et al., 2013; Tsiaras et al., 2011) and ADHD (Ahmadlou, Adeli, & Adeli, 2012).

While these connectivity and graph measures show potential as biomarkers to detect atypical development, biomarkers are only useful if they have a low inter‐subject variability and a high test‐retest reliability (Hardmeier et al., 2014). Several studies have shown that this is the case for adult EEG/MEG networks (Deuker et al., 2009; Hardmeier et al., 2014; Kuntzelman & Miskovic, 2017). Whether this also holds true for infants, however, is currently unknown. For the early detection of neurodevelopmental disorders, it is especially vital that network measures are reliable during infancy. Therefore, in this study, we set out to determine the test‐retest reliability and inter‐subject variability for functional EEG network measures, created by task‐dependent continuous EEG in infants.

## 2 METHODS & MATERIALS

### 2.1 Subjects & Procedure

Seventy‐seven 10‐month‐old infants, recruited from communal registers in the Netherlands, participated in the study. The final sample consisted of 60 infants (29 males, at first visit: mean age = 301 days, range = 272–342, at second visit: mean age = 308 days, range = 279–349). During the EEG recording infants were seated in a high chair and watched 2 different one‐minute videos on a computer screen, three separate times. The first video depicted social stimuli with singing women as the subject, the second video depicted non‐social stimuli of toys that were moving without human interference, earlier used in a study by Jones and colleagues (Jones, Venema, Lowy, Earl, & Webb, 2015). The lack of fixed sleep patterns in most young infants, caused the start times of the experiment not to be fixed over sessions. However, where possible, infants were tested before noon. Eat and sleep patterns were recorded on both sessions. These patterns could not be kept similar across sessions*. *The parents/guardians received information about the study beforehand and signed an informed consent form before the start of the first session. The medical ethical committee of the University Medical Center Utrecht approved the study (application number: 14‐221). Children received a toy after participation.

### 2.2 EEG acquisition

EEG was recorded using a cap with 32 electrodes (ActiveTwo system, BioSemi) positioned according to the international 10/20 system, at a sampling rate of 2048 Hz. A Common Mode Sense (CMS) and Driven Right Leg (DRL) electrode were used to provide an active ground. In addition, two mastoid electrodes (EXG1 & EXG2) were placed behind the ears and one ocular electrode under the eye (EXG3).

### 2.3 EEG analysis

EEG data were analyzed exclusively using Matlab, by means of the FieldTrip toolbox (Oostenveld, Fries, Maris, & Schoffelen, 2011). The original 2048 Hz data were down sampled to 512 Hz, using chip interpolation and band‐pass filtered at 0.1–70 Hz with a two‐way Butterworth filter. Data were purposefully not deep cleaned, to limit subjective outside influences pushing the data into a highly reliable mold. However, clearly nonneurological signals, like jumps, cuts, and high variability within a trial, were detected and removed. Channels were removed if more than 50 percent of the signal in a channel contained artifacts. Bad channels were removed from both sessions of a subject. The cleaned data were used for further analysis.

### 2.4 Connectivity calculation

The cleaned data for each subject were bandpass filtered into six bands: delta (0.5–3 Hz), theta (3–6 Hz), alpha1 (6–9 Hz), alpha2 (9–12 Hz), beta (12–25 Hz), and gamma (25–45 Hz). Since individual theta and alpha peaks are influenced by development, alpha1, and theta bands were chosen to encompass all theta and alpha peaks ±1 Hz. The resulting data were cut into 5s. epochs. Twenty random epochs were picked per subject per session. For each epoch, connectivity between pairs of electrodes (32*31/2 = 496) was calculated with the phase lag index (PLI) and the debiased weighted PLI, both relying on the same principle of phase locking or phase synchrony (Tass et al., 1998). The PLI, proposed by Stam et al., (Stam, Nolte, & Daffertshofer, 2007), describes the asymmetry of the distribution of phase differences between pairs of signals:

PLI=|sign[sin(Δφ(tk))]|

where Δφ is the instantaneous phase difference between signals at time point *t* for *k* = 1 … N per epoch (*N* = 5*512 = 2,560), determined using the Hilbert transformation. || stands for absolute values, <> for the mean values and the sign for a signum function (phase difference is either −1, 0, or 1). The resulting PLI can range from 0 to 1. Volume conduction, the effect that multiple electrodes register activity from the same source, plays a minimal role in the PLI. Activity from a single source will appear in both electrodes as having a phase difference of exactly zero. Since the PLI indexes the stability of phase leaping or lagging, a phase difference of zero will lead to a PLI of zero.

The debiased weighted PLI (dwPLI) is an adjustment of the PLI developed by Vinck and colleagues (Vinck, Oostenveld, van Wingerden, Battaglia, & Pennartz, 2011). The PLI is weighted by the amount of lag between the two signals, thereby limiting the influence of near zero phase differences. This minimizes the amount of false positive connectivity between near zero phase difference signals, which could be caused by noise in the data. Since infant data are notorious for its noisiness, the dwPLI is included as well. Our used version of the weighted PLI also debiases the connectivity based on the number of epochs, since infant data likely involve few trials. This debiasing can cause the dwPLI to be negative and, therefore, ranges from −1 to 1.

### 2.5 Graph analysis

Several graph measures were calculated using the acquired individual connectivity matrices. The complete weighted matrices were used, eliminating the need for arbitrary thresholds. The following graph measures were calculated using the brain connectivity toolbox (Sporns & Rubinov, 2010) (Table 1): average clustering coefficient (Cw), characteristic (average shortest) path length (Lw); and small‐worldness index (SWI, calculated as the ratio between normalized Cw and normalized Lw). Both the averaged clustering coefficient and the characteristic path length are normalized to limit the influence of global connectivity on these characteristics.

**Table 1:** Graph measures references and formulae

Name		Formula	Reference
Average clustering coefficient	Cw	$\text{Cw}=\frac{1}{n}\sum_{i\in N}\frac{2t_{i}}{k_{i}\left(k_{i}-1\right)}$	Onnela, Kaski, and Kertész (2004)
Characteristic path length	Lw	$\text{Lw}=\frac{1}{n}\sum_{i\in N}\frac{\sum_{j\in N,i\neq j}d_{\mathrm{ij}}}{n-1}$	Watts and Strogatz (1998)
Small‐worldness Index	SWI	$\text{S}=\frac{\text{C}/{\text{C}}_{\text{rand}}}{\text{L}/{\text{L}}_{\text{rand}}}$	Humphries and Gurney (2008)

### 2.6 Statistical analysis

**Figure 1:**
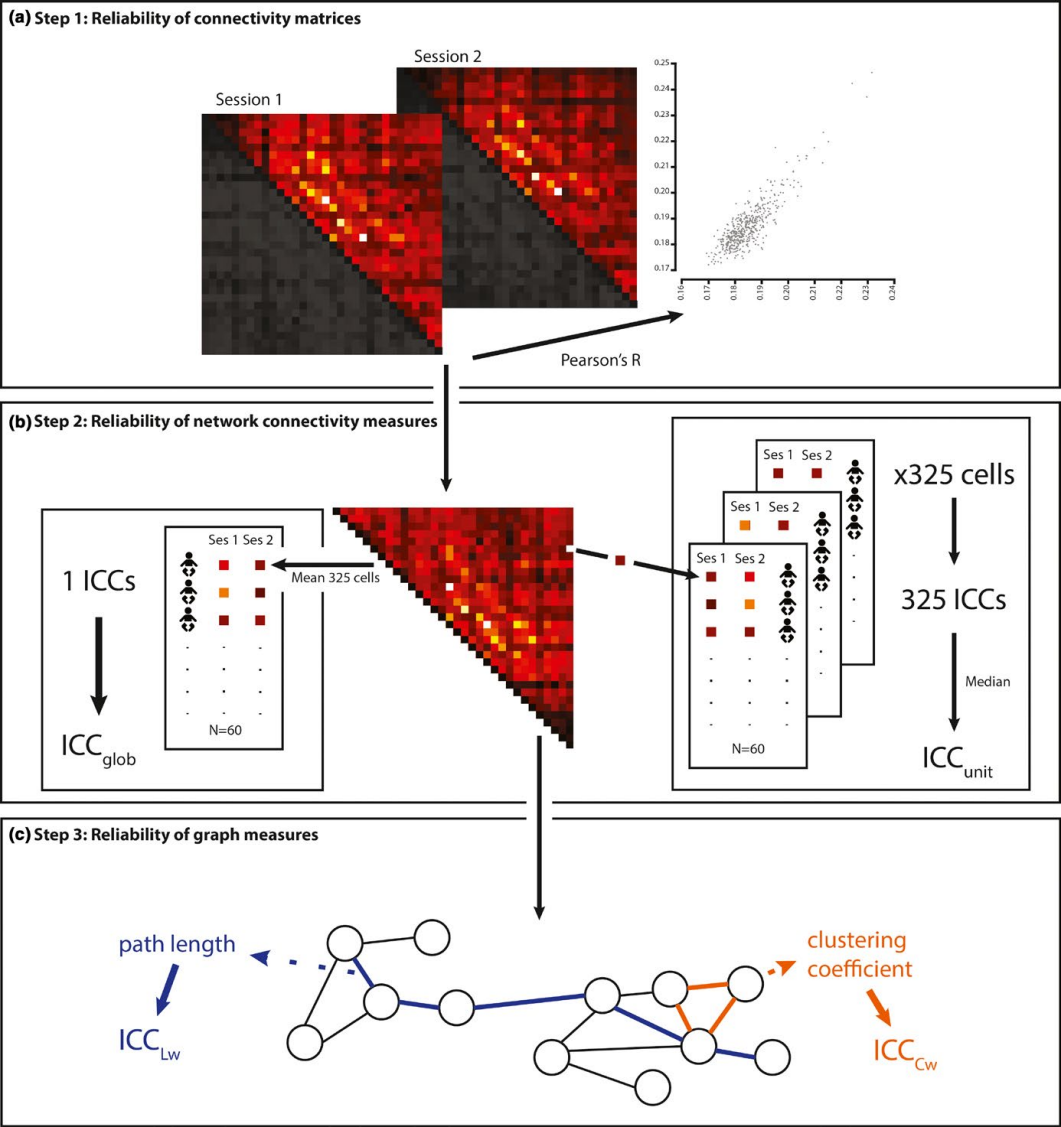
Overview of the different steps in network analysis and their respective reliabilities. This figure shows the complete steps of network analysis and graphically depicts the reliabilities calculated for each step. (a) reliability at the most fundamental level, in which connectivity matrices are correlated over sessions for each subject, for each frequency band. (b) reliability of global (left) and local, “unit‐wise” (right), connectivity. (c) graph theoretical representation of the network and several graph characteristics, which are compared over sessions

The test‐retest reliability was determined differently across three different steps of the analysis (Figure 1). At the most basic level (step 1, Figure 1a), the complete connectivity matrices were compared over sessions by calculating the Pearson's correlation coefficient (R). The reliability of the connectivity measures of steps 2 and 3 (Figure 1b,c) were calculated by comparing sessions through an intra‐class correlation (ICC) (Shrout & Fleiss, 1979; Weir, 2005), which uses a one‐way ANOVA to determine the mean squared error (${\text{MS}}_{\text{e}}$) and the between object (subject) variance (${\text{MS}}_{\text{r}\;}$). Shrout and Fleiss (1979) describe six distinct statistical models which carry the name, of which we are using an ICC(3,1) two‐way mixed effect model, similar to other studies on the reliability of graph measures (Hardmeier et al., 2014; Hatz et al., 2016). ICC values were calculated using:

$$\text{ICC}=\frac{{\text{MS}}_{\text{r}\;}-{\text{MS}}_{\text{e}}}{{\text{MS}}_{\text{r}\;}+\left(\text{k}\;-1\right){\text{MS}}_{\text{e}}}$$

where *k* is the number of measurements per subject.

We assessed the reliability of both global and local (dw)PLI connectivity matrices (step 2, Figure 1b). The global PLI/dwPLI (ICC_glob_) was calculated by averaging over all 325 electrode pairs of each subjects’ matrix, creating one value per subject per frequency band per session. A single ICC value per frequency band was calculated by comparing session 1 versus session 2. The local PLI/dwPLI unit‐wise reliability was determined by calculating an ICC value per electrode pair over all subjects’ session 1 versus session 2, creating 325 ICC values. Since these values did not follow a normal distribution, the median was taken as the single reliability value (ICC_unit_). To summarize, the reliability of the global PLI/dwPLI is the reliability of all connections combined, while the unit‐wise reliability is the median reliability of all the reliabilities of individual connections. To test the influence of noisy connections with low connectivity, an average connectivity matrix was calculated based on all connectivity matrices from both sessions. The top 25th percentile of connections were selected based on connectivity strength and the unit‐wise reliability calculation was performed using only these connections for each subject (Guo et al., 2012).

To test the reliability of the graph measures (Cw, Lw, and SWI), values were calculated for each subject, per session, per frequency band (step 3, Figure 1c). An ICC was used to calculate the reliability of these graph measures over sessions. In accordance to previous research on graph metrics, we report ICC values below 0.4 as low reliability, 0.4 < ICC < 0.6 as mediocre reliability, 0.6 < ICC < 0.75 as good reliability and an ICC >0.75 as excellent reliability (Hardmeier et al., 2014; Jin, Seol, Kim, & Chung, 2011). To understand the effect of outliers, a bootstrapping procedure with replacement and 10,000 permutations was used to estimate the 95% confidence intervals for both COV and ICC values, similarly used by Hardmeier and colleagues (Hardmeier et al., 2014). For a clear overview of the reliability tests, please refer to Figure 1. Lastly, for both the connectivity and graph measures the inter‐subject variability was determined using the coefficient of variation (ratio between mean and standard deviation).

It is common to perform spectral analyses along with the connectivity analyses to get a better overview of how power and connectivity are associated. Therefore, reliability of EEG‐power metrics was calculated as well. Results and methods for this section can be found in Data S1.

## 3 RESULTS

### 3.1 Reliability of connectivity matrices

**Figure 2:**
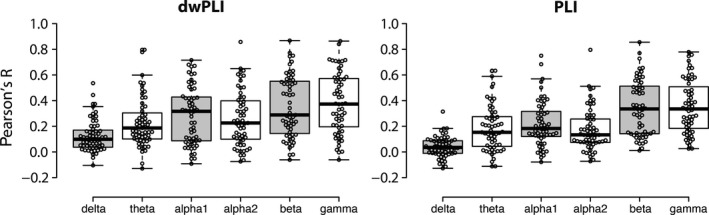
Connectivity matrix correlation coefficients for all frequency bands. Boxplot of all individual connectivity matrix correlations for session 1 versus session 2, shown for delta, theta, alpha1, alpha2, beta, and gamma. The left graph shows the correlation coefficients for the connectivity matrices calculated with the dwPLI, the right graph shows the PLI calculated connectivity matrices. Correlations range widely, but the median of the correlations within each frequency band is low. Plotted with BoxplotR (Spitzer, Wildenhain, Rappsilber, & Tyers, 2014)

The results of the correlation of the connectivity matrices across sessions are presented in Figure 2. Correlation coefficients range widely and the median of the coefficients is generally low. There is little difference between the reliability of dwPLI and PLI connectivity matrices, showing ranges of respectively 0.1–0.37 and 0.03–0.33.

### 3.2 Reliability of network connectivity measures

Inter‐subject variability (Table 2) of global PLI was low for all frequency bands (0.02 < COV_glob_ < 0.12). Reliability of global PLI (Table 3) was excellent for theta, alpha1, and alpha2 frequency bands (0.84 < ICC_glob_ < 0.91), mediocre to good for delta, gamma and beta frequencies (0.60 < ICC_glob_ < 0.72). Global PLI values at session 1 differed significantly between frequencies (F: 772, *p* < 0.00001). All frequency bands differed significantly from each other (*p* < 0.00001), except for alpha1 and alpha2 global connectivity values (*p* = 0.11).

**Table 2:** Inter‐subject variability of global connectivity with 95% confidence intervals

	Delta	Theta	Alpha1	Alpha2	Beta	Gamma
PLI	0.02	0.12	0.09	0.05	0.05	0.05
95% CI	0.02–0.03	0.09–0.15	0.08–0.11	0.02–0.06	0.04–0.06	0.03–0.06
dwPLI	0.92	1.11	0.73	1.15	0.76	0.91
95% CI	0.47–1.18	0.89–1.33	0.60–0.89	0.64–1.42	0.60–0.89	0.64–1.11

**Table 3:** Test‐retest reliability of global connectivity with 95% confidence intervals

	Delta	Theta	Alpha1	Alpha2	Beta	Gamma
PLI	0.60	0.91	0.84	0.86	0.72	0.61
95% CI	0.38–0.73	0.82–0.95	0.71–0.92	0.61–0.93	0.52–0.83	0.32–0.74
dwPLI	−0.29	0.82	0.75	0.91	0.74	0.49
95% CI	−1.51–0.11	0.69 – 0.90	0.54–0.87	0.39–0.97	0.47–0.88	0.28–0.73

Compared to global PLI, inter‐subject variability of global dwPLI (Table 2) was higher (0.91 < COV_glob_ < 1.15) and reliabilities were lower, with theta, alpha1, alpha2 frequencies showing good to excellent reliability (0.75 < ICC_glob_ < 0.91) and delta, beta, and gamma frequencies having a poor reliability (−0.29 < ICC_glob_ < 0.74) (Table 3). Also, note the wider 95% confidence intervals for the dwPLI calculated global connectivity. Therefore, dwPLI is excluded from this point onwards in the results to prevent misinformation.

**Figure 3:**
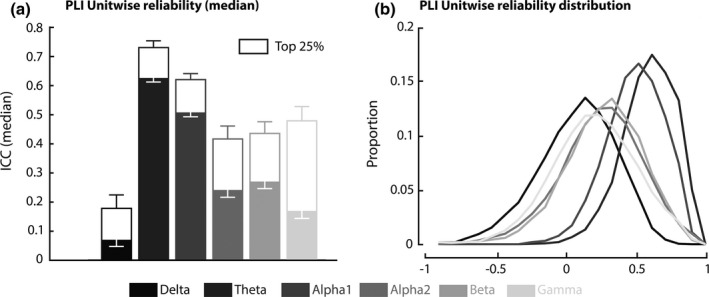
Reliability of unit‐wise PLI. (a) Bar plot of ICC values for unit‐wise reliability per frequency band with theta and alpha1 showing the highest reliability. All frequency bands show marked improvement when only the on average strongest 25% of connections are used. Errorbars represent 2SE. (b) Distribution of ICC values for all frequency bands. All frequency bands show a distribution skewed towards the positive ICC values. This is most pronounced in the theta and alpha1 frequency band

The reliability of local, unit‐wise, PLI connectivity was lower than global PLI, with the median ICC showing mediocre to good reliability in the theta and alpha1 frequency band (0.50 < ICC_unit_ < 0.62) and delta, alpha2, beta and gamma frequency bands showing poor reliability (0.07 < ICC_unit_ < 0.27). The reliability of unit‐wise connectivity improved considerably when using the 25th top percentile of the on average strongest connections over both sessions (Figure 3a), with both theta and alpha1 having good reliability (0.62 < ICC_unit_ < 0.73), alpha2, beta and gamma having mediocre reliability (0.41 < ICC_unit_ < 0.48). Delta local connectivity reliability is still poor (ICC_unit_ = 0.18). The distribution of unit‐wise reliability showed a considerable spread in ICC values. All frequency bands were skewed towards the higher ICC values, with alpha1 and theta frequency bands being most pronounced (Figure 3b).

### 3.3 Reliability of graph measures

**Figure 4:**
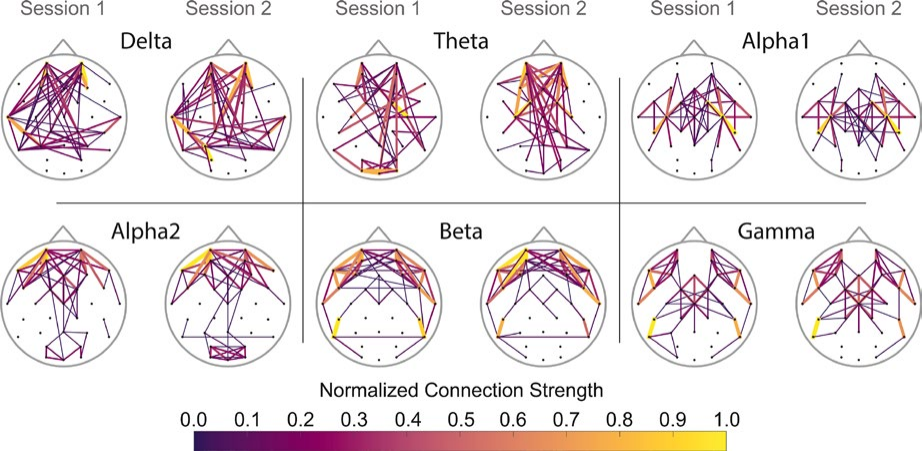
Average connectome per frequency band per session. The averaged connectomes with the 12 percent strongest connections show great similarity between sessions. Yellow, thicker lines depict higher normalized connection strength. Blue, thinner lines depict lower normalized connection strength. Note the higher dependency on long range connections in slower oscillations

Table 4 shows the reliability of graph measures calculated from the PLI matrices. PLI average clustering coefficient (Cw) was excellently reliable across alpha1, alpha2 and theta frequency bands (0.84 < ICC_Cw_ < 0.91) and was mediocre to good in reliability in delta, beta and theta frequency bands (0.59 < ICC_Cw_ < 0.73). Lw_nrm _showed excellent reliability across theta, alpha1, apha2, and gamma frequency bands (0.84 < ICC_Lw_ < 0. 89) and mediocre to good reliability in delta, theta, and beta frequency bands (0.53 < ICC_Cw_ < 0.72). The small‐worldness index (SWI) was least reliable, with mediocre reliability in theta and alpha1 frequency bands (0.56 < ICC_SWI_ < 0.67) and poor reliability in the delta, alpha2, beta, and gamma frequency bands (0.13 < ICC_SWI_ < 0.25. During session 1, not all networks showed small‐worldness (range: 0.9869 < SWI < 1.02). Average connectomes were created for both sessions for all frequency bands, which shows a strong similarity in strongest connections and connection strength between session 1 and 2 (Figure 4).

**Table 4:** ICC reliability of PLI graph measures

	Delta	theta	alpha1	alpha2	beta	Gamma
Cw_nrm_	0.59	0.91	0.84	0.87	0.73	0.62
95% CI	0.32–0.73	0.81–0.95	0.81–0.91	0.63–0.93	0.53–0.84	0.37–0.75
Lw_nrm_	0.53	0.89	0.84	0.84	0.72	0.59
95% CI	0.19–0.71	0.79–0.94	0.72–0.92	0.63–0.92	0.53–0.84	0.33–0.75
SWI	0.25	0.56	0.67	0.21	0.14	0.13
95% CI	−0.02–0.54	0.36–0.73	0.40–0.83	−0.47–0.71	−0.07–0.34	−0.10–0.49

## 4 DISCUSSION

In this paper, we showed for the first time that infant functional brain network characteristics can be reliable, by determining the test‐retest reliability and the inter‐subject variability of infant functional EEG connectivity across a 1‐week period. Overall, reliabilities of global connectivity characteristics were high, while more local characteristics showed lower, though still acceptable reliabilities. Characteristics calculated with the connectivity matrices of theta and alpha1 frequency bands were most reliable. This pattern of reliability is similar to earlier studied reliability of adult network characteristics.

Broadly, the reliability of EEG networks can be assessed on three levels, which coincide with three steps of network analysis: The reliability of (a) the complete connectivity matrices, (b) global and local functional connectivity measures gathered from these matrices and, (c) graph characteristics gathered from the graphs created from these matrices. Firstly, we reported that connectivity matrices correlate poorly over sessions. Reliabilities of complete connectivity matrices have, to our knowledge, never been reported for EEG networks in adults nor infants. It is thus difficult to compare our reliabilities to other studies. Most other studies focus on the reliability of steps two and three of connectivity analysis: global and local connectivity measures; and graph characteristics.

Secondly, we found excellent test‐retest reliability of global connectivity, the average of all connections in a connectivity matrix. Local, unit‐wise, reliability showed a wide range of test‐retest reliabilities across most frequency bands, ranging from connections with very low reliability to connections with excellent reliability. This is in concurrence with several adult MEG/EEG reliability studies. Hardmeier and colleagues reported excellent global connectivity reliability in their eyes‐closed resting state EEG study, in theta, alpha1, and alpha2 frequency bands, while local inter‐regional connectivity ranged from poor to excellent across all frequency bands (Hardmeier et al., 2014). Deuker and colleagues found good test‐retest reliability for MEG global connectivity during eyes‐open resting state and excellent reliability during an n‐back task in theta and alpha frequency bands (Deuker et al., 2009). Lastly, Jin and colleagues found moderate to high test‐retest reliability in eyes‐open and closed MEG resting state global connectivity, in theta and alpha frequency bands (Jin et al., 2011).

Thirdly, the reliability of global first order graph metrics tested in this study ranged from moderate to excellent, with both average clustering coefficient (Cw) and characteristic path length (Lw) being excellently reliable across theta, alpha1, and alpha2 frequency bands. This is also found in other EEG network reliability studies. Previously mentioned Hardmeier and colleagues also tested the reliability of graph metrics and found excellent reliabilities for both Cw and Lw in theta, alpha1 and alpha2 bands (Hardmeier et al., 2014). More recently, Kuntzelman & Miskovic tested adults during an eyes‐closed resting state EEG paradigm, comparing global and local graph measures on coherency and dwPLI. They reported good reliability of global dwPLI metrics in theta, alpha1 and alpha2 frequency bands (Kuntzelman & Miskovic, 2017).

Across the study, we report lower reliabilities for delta, beta and gamma frequency bands than for theta, alpha1, and alpha2 frequency bands. This is in concurrence with several previously mentioned studies in which lower beta and gamma reliabilities (Hardmeier et al., 2014; Jin et al., 2011; Kuntzelman & Miskovic, 2017); and lower delta reliabilities (Deuker et al., 2009; Kuntzelman & Miskovic, 2017) were found. Most commonly, the lower reliability of higher frequency bands is explained by the dichotomy between higher and lower frequency bands, where higher frequency bands are more involved in establishing cognitive representation, while lower frequencies are more anatomically constrained (Bassett & Bullmore, 2006). This constraint could aid higher reliabilities over sessions. Also, both theta and alpha have been suggested to be important for processing attention (Aftanas & Golocheikine, 2001; Klimesch, Doppelmayr, Russegger, Pachinger, & Schwaiger, 1998) and top down control (Engel, Fries, & Singer, 2001). Since our task could specifically target these systems, the resulting higher signal to noise ratio in these frequency bands could result in more reliable networks. Lastly, the higher prevalence of muscle artifacts in the higher frequency bands could limit reliability, especially in children. The small‐worldness index (SWI) is also less reliable in our study, which is in concurrence with previous studies (Hardmeier et al., 2014; Kuntzelman & Miskovic, 2017). Since small‐worldness is calculated using both clustering coefficient and path length, and both these characteristics vary independently across sessions, a combination of these variances in the SWI (SWI) could contribute to a lower reliability for the SWI.

The overall spatial resolution has a large influence on test‐retest reliability with global connectivity characteristics being highly reliable, while local connectivity characteristics are somewhat less reliable. This study also shows that different steps of the analysis yield different reliabilities. Most interestingly, lowly reliable connectivity matrices generate highly reliable connectivity and graph characteristics, which can be explained in several ways. Firstly, it is possible that some lowly connected, noisy connections are present in the full connectivity matrices, which are averaged out in global connectivity characteristics. Secondly, brain networks fluctuate in activity over time (Chang & Glover, 2010). It is possible that, comparing multiple sessions, the state of the network is different, but the underlying characteristics and anatomy are equal. Thirdly, a difference in fixing the EEG cap over sessions could lead to a rotation in connectivity matrices over sessions (Hatz et al., 2016) and lastly, an unknown covariate, that remains stable over sessions, could influence network characteristics, but not connectivity matrices. It is currently unknown which of these explanations (or a combination of these explanations) is correct and future research is needed to further understand the relationship between unreliable connectivity matrices and reliable connectivity characteristics.

It is important to note that reliability does not imply validity and that this study, therefore, does not allow conclusions on the validity of these measures. It is currently unknown how tightly these measures reflect true cortical and subcortical brain connectivity. This becomes more difficult with EEG, which is restricted to measuring activity at the sensor level. While resting state oscillations have been found to be connected to resting‐state connectivity gathered from functional MRI data (Laufs, 2008; Mantini, Perrucci, Del Gratta, Romani, & Corbetta, 2007), in our study, due to the difficulty of doing resting‐state research with infants, we opted for a continuous video stimulus. While this makes it more difficult to understand how these network characteristics are reflected in the structural connectome, it comes with the added benefit of minimizing the variance over sessions, thereby possibly improving reliability. This is also reflected in the study by Deuker and colleagues, where task‐dependent connectivity measures were shown to be more reliable than resting state connectivity measures (Deuker et al., 2009). In addition, previous research has shown the influence of global connectivity on both characteristic path length and average clustering coefficient. Therefore, the high reliability of both these metrics in this study could be explained through the high reliability of global connectivity. Even normalizing these graph metrics does not completely eradicate this problem and future research is therefore necessary to understand the exact implications of this (van den Heuvel et al., 2017).

While the validity of these measures can be disputed, previous research has shown the potential of network characteristics as biomarkers of neurodevelopmental disorders. Orekhova and colleagues found that while comparing infants at risk for ASD, global connectivity was related to whether or not an infant actually developed ASD (Orekhova et al., 2014). Boersma and colleagues found similar results when comparing toddlers with ASD to toddlers without ASD (Boersma et al., 2013). Others have noted differences in graph characteristics in adults suffering from ASD (Belmonte et al., 2004) and ADHD (Ahmadlou et al., 2012). This, together with the here reported excellent reliability of graph and connectivity measures in theta, alpha1 and alpha2 frequency bands in infants, underlines the potential of using these measures to detect neurodevelopmental disorders at an earlier age, conceivably increasing our fundamental knowledge on how these disorders develop and could possibly be treated.

## 5 CONCLUSIONS

This study showed for the first time that global and to a lesser extent local PLI connectivity measures in infants are reliable over a 1‐week period. We recorded EEG from infants twice, one week apart, while they were watching social and nonsocial videos. We found that when comparing the resulting PLI networks, global network measures are stable over time. Reliable global network measures could play a vital role in finding biomarkers for several disorders. The unrestrictive nature and the relative ease of an EEG recording make it especially useful to detect these network characteristics at a very young age, giving us important insight in the development of these disorders, possibly making early detection, and intervention possible.

## CONFLICT OF INTEREST

The authors have no conflict of interest do declare.
