# Supplementary material for: Test‐retest reliability of EEG network characteristics in infants
Source: Brain Behav. 2019 Mar 25;9(5):e01269. doi: 10.1002/brb3.1269 (PMC6520303; doi:10.1002/brb3.1269)
Supplement: Supplementary file 1 [file BRB3-9-e01269-s001.docx]

## Supplementary methods and data

It is common to perform spectral analyses along with the connectivity analyses to get a better overview of how power and connectivity are associated. Therefore, reliability of EEG-power metrics was calculated as well. We used the cleaned data used for the reliability calculation of connectivity measures. A multitaper time-frequency analysis, implemented with the FieldTrip toolbox (Oostenveld, Fries, Maris, & Schoffelen, 2011), was performed on the signal from each EEG channel using a moving window of 2s with an overlap of 50%. Of the resulting power distributions per channel per subject, area under the curve (AUC) was calculated for each frequency band. The reliability of these AUCs was calculated using the same ICC-method as described above. Comparing each AUC for each channel over sessions.

The main results are congruent with the earlier found reliability for connectivity measures, with most channels in the theta, alpha1, and alpha2 frequency bands being extremely reliable (median > 0.8). Beta and gamma frequency bands showing highly varying reliability across channels and the delta frequency band showing mostly unreliable power in all channels. Results are summarized in supplementary figure 1 and supplementary figure 2. Lastly, the reliability of the full frequency distribution
(between 0.5 and 45 Hz.) averaged over all electrodes was estimated, which shows clearly higher reliabilities around in the theta and alpha frequency bands (see supplementary figure 3).

**Supplementary figure 1. Reliability of area under the curve for different frequency bands.** Violinplot depicting the reliability of the area under the curve of the power distribution of all channels (32) separated in delta, theta, alpha1, alpha2, beta, and gamma frequency bands. Every dot represents the reliability of an individual channel in a specific frequency band.


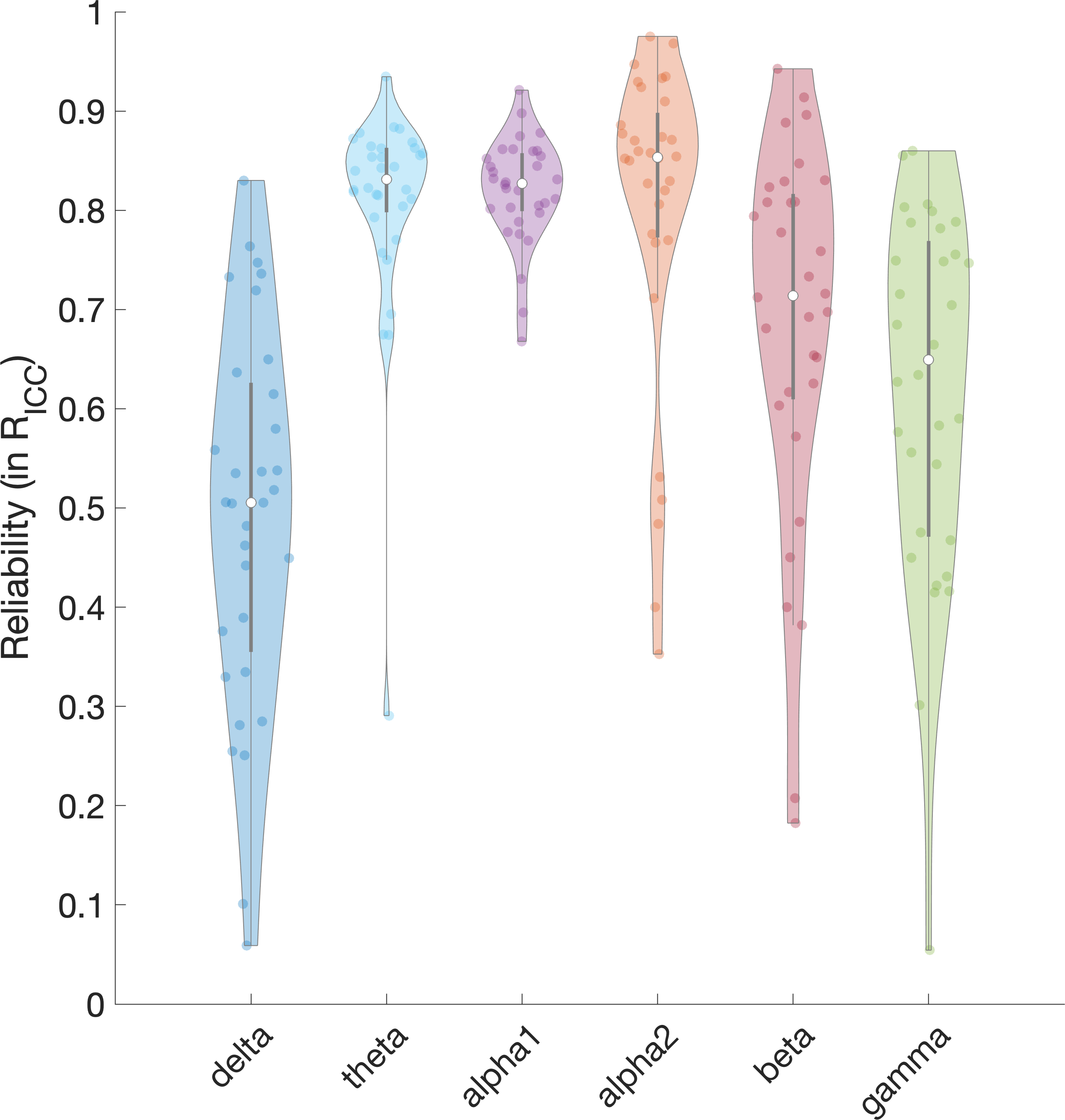


**Supplementary figure 2. Reliability of area under the curve for individual channels.** Topoplot representing the reliability of area under the curve of the power distributions. Yellow indicates higher reliability and purple/blue indicates lower reliability. Theta, alpha1, and alpha2 frequency bands showing high reliability throughout most electrodes. Beta and gamma frequency bands showing highly variable reliability, with occipital electrodes generally showing higher reliability and frontal electrodes lower reliability. The delta frequency band is generally unreliable.


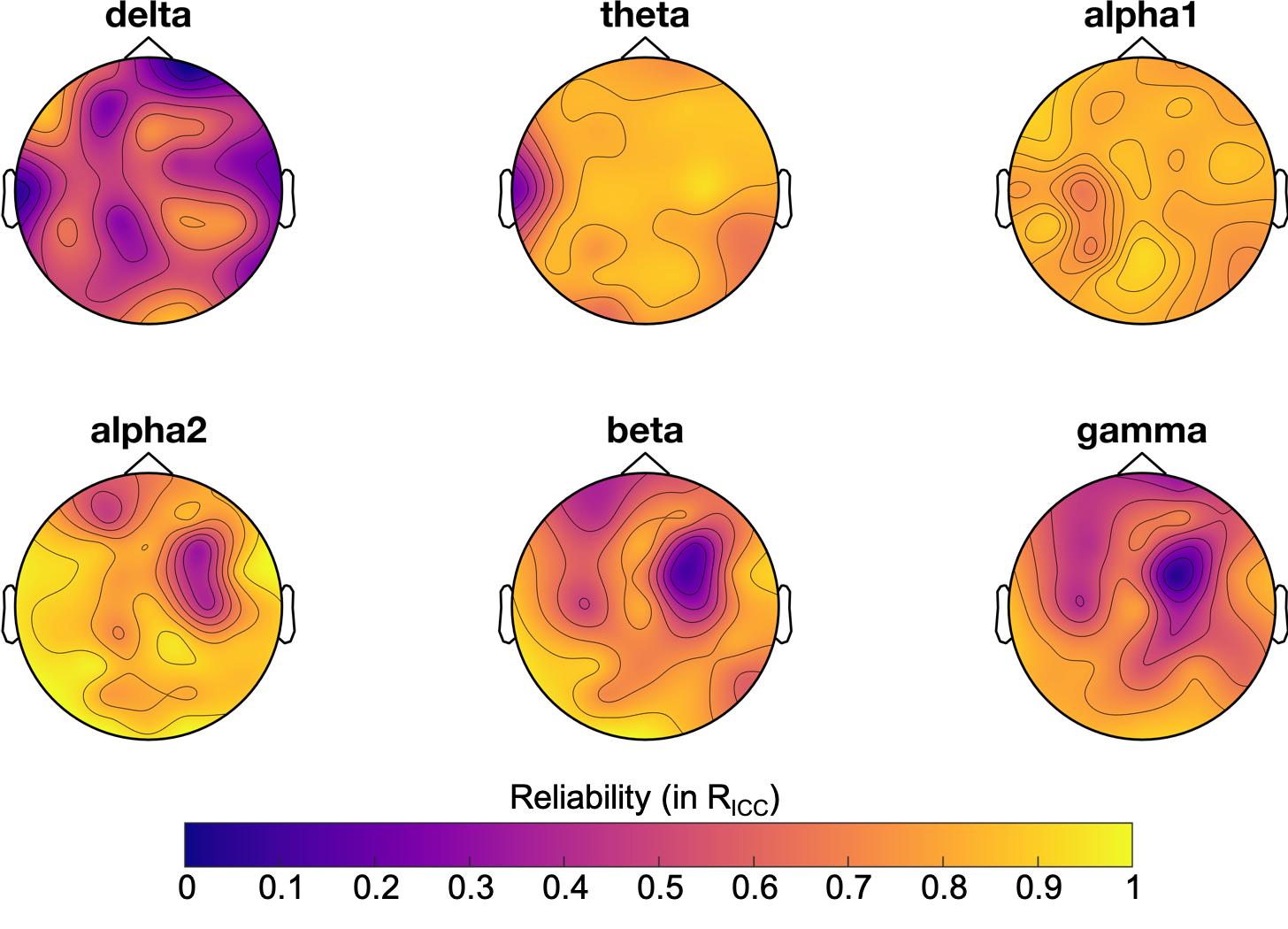


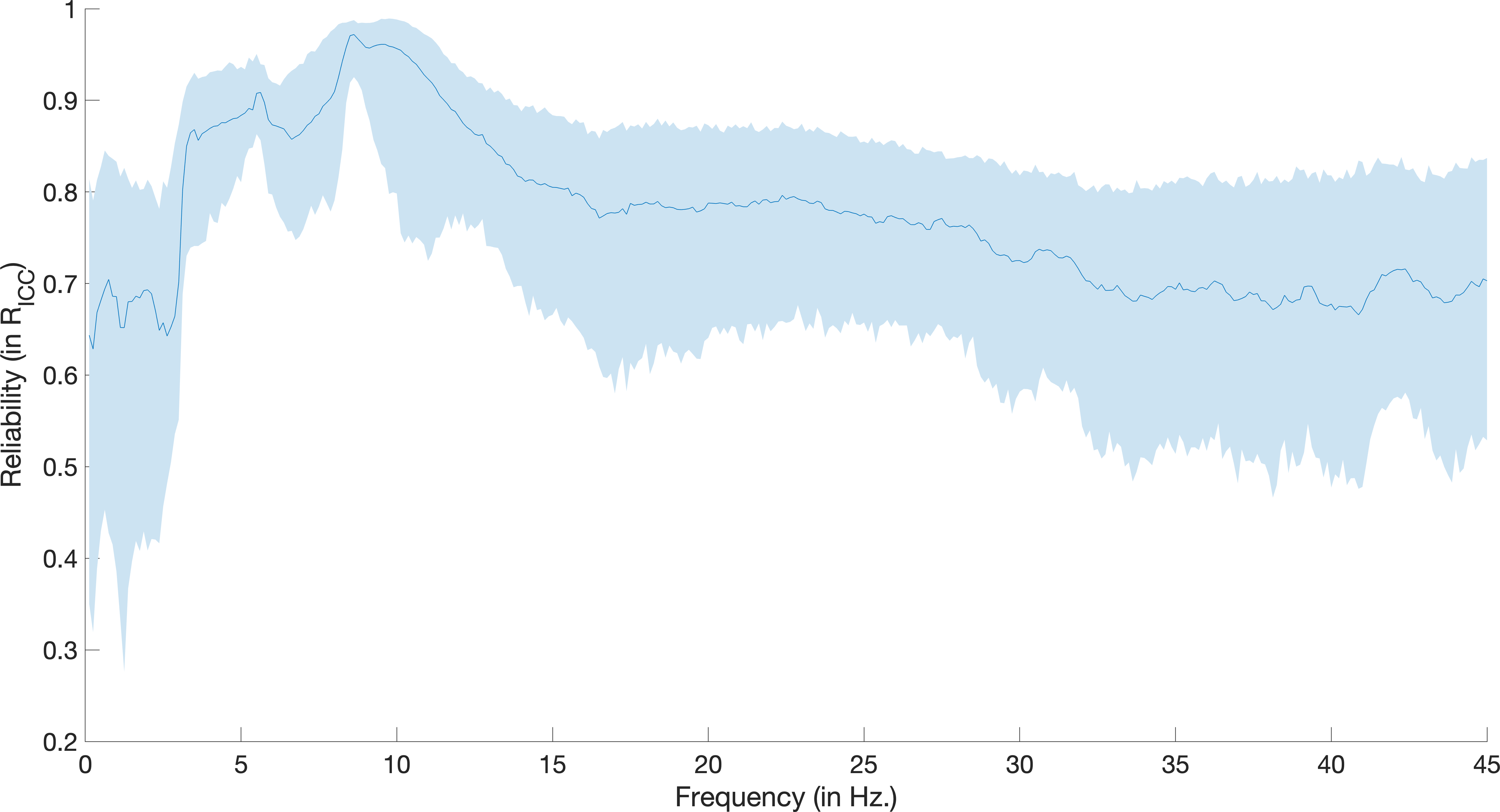


**Supplementary figure 3. Reliability of frequency distribution.** The dark blue line represents the reliability of every single point of the frequency distribution averaged over all electrodes. Light blue shading shows the 95% confidence interval of this reliability value, estimated with a bootstrapping procedure with 10000 permutations. Clear peaks in reliability can be seen around the theta (3 - 6 Hz.) and alpha (6 – 12 Hz.) frequency bands.
